# The Comanche Co. Medical Society

**Published:** 1885-07

**Authors:** 


STILL ANOTHER.—The "Comanche County Medical Society" has been organized with Dr. G. W. Tucker President, and Dr. C. F. Paine Secretary.

